# Reading comics: The effect of expertise on eye movements

**DOI:** 10.16910/jemr.17.4.5

**Published:** 2024-11-15

**Authors:** Hong Yang

**Affiliations:** School of Literature and Journalism Sichuan University, China

**Keywords:** Eye tracking, Comic, Visual Literacy, The Theory of Expertise

## Abstract

The theory of expertise suggests that there should be observable differences in the eye movement patterns between experts and non-experts. Previous studies have investigated how expertise influences eye movement patterns during cognitive tasks like reading. However, the impact of expertise on eye movements in comics, a multimodal form of text, remains unexplored. This article reports on a study that uses eye tracking to examine patterns in the ways that experts and non-experts read comics. Expert participants (14) with experience in reading comics than non-expert participants (17). When controlling for variables such as layout and text quantity, we found significant differences in visual scanning between experts and non-experts. Experts exhibited more frequent saccades and greater amplitude of saccades. Further analysis revealed distinct strategies in processing text and image content between the two groups: the interaction between expertise level and content type in specific AOI showed significant differences across multiple visual measurement metrics, including Average duration of fixations, number of fixations, and number of saccades within AOI. These findings not only support the applicability of the expertise level theory in the field of comic reading but also provide a new perspective for understanding the reading processing of multimodal texts.

## Introduction

### Eye tracking research on visual expertise

In the era of visual media, the significance of visual literacy is on the rise. Experts exhibit faster and more accurate identification in their fields (14), efficiently pinpointing key features and employing advanced parafoveal processing, unlike novices (25).

As key proponents of the theory of expertise, Ericsson and Charness (9) emphasize that deliberate practice is the crucial factor in expert performance, explaining the mechanisms behind the formation of expertise. The study of expertise encompasses a broad spectrum of fields, including sports, music, chess (37), and medicine (23). Expertise in different areas affects task performance in various ways and is generally considered to be domain-specific. Some researchers have pointed out that expert performance across different fields shares common characteristics (see 38), such as employing “chunks” and “templates” to accurately represent highly complex search patterns (25).

The mind-eye hypothesis, as proposed by Just and Carpenter (16), suggests that gaze behavior reflects underlying cognitive processes. Research on eye movements in reading and in scene perception also assume that fixation is indicative of visual attention (36).

Eye tracking provides insight into physical viewing behaviors and the cognitive processes behind these behaviors. We can identify experts based on eye tracking data (20). Eye movements are indicators of visual attentional processes (5). Lesgold and colleagues (24) were among the pioneers in using eye-tracking studies to explore visual expertise within the intricate realm of medical practice.

Research confirms that experts and non-experts exhibit distinct eye-fixation patterns in art (1) and reading (36). When examining art, experts employ a unique visual scanning strategy, characterized by a heightened sensitivity to high-level features. These features include textures and the composition of colors (19), overall composition (11), and structural features (42). Eye movement patterns, such as dwell time, average fixation duration, and fixation count, differ based on the viewer's expertise (4; 11; 41). When examining reading, A high-level speed-reading expert characterized by nearly straight horizontal eye movements during the first pass of reading (30). The perceptual span of beginning readers is smaller than that of skilled readers (36; 40).

Kristjanson and Antes (21) and Antes and Kristjanson (1) observed distinct viewing patterns between artists and non-artists when looking at known and unknown paintings. Artists showed higher fixation density and shorter average duration of non-central fixations on unknown paintings, compared to lower fixation density but longer average duration of non-central fixations on known paintings. This highlights the importance of considering the familiarity of the materials in the study of viewing behavior.

Various research studies have focused on the reception of artwork, encompassing paintings, sculptures, advertisements, music reading, and museum (27; 41; 42). However, less attention has been given to the understanding of multimodal texts such as cartoons and graphic novels (39), despite their growing popularity among the digital generation.

While the impact of expertise on visual processing is well-established across diverse fields like reading (36) and medicine (23), its manifestation in the subtler domain of everyday reading, particularly in multimodal contexts, remains underexplored. Most of these studies have tended to focus on visual search tasks, while rather neglecting reading in actual environments. There's a notable lack of research on eye movement patterns in interpreting multimodal reading like comics and graphic novels, journalistic photographs (3; 4), and music reading (33).

### Eye tracking on comic reading

Many comic researchers attempt to explain how comics communicate and create meaning, focusing on perspectives such as narrative structure and semiotics. Notable contributions include Postema's analysis on narrative structures in comics (34) and Wildfeuer's exploration of comic semiotics (45).

Some scholars have adopted empirical research paradigms to understand comics. They utilized eye trackers to analyze fixation patterns in comic reading and discussed factors in comic stimuli guiding eye movements (32). This includes the visual sequence of a comic strip (10), the structure of comic panels (6), the external structure (outlines) of panels and panel content (18), and onomatopoeia (39).

Laubrock et al. (22) discovered that text in comic panels attracted more attention than images, even though the text areas usually occupy much less space in the panels than the images do. Similarly, Kirtley et al. (18) found that the presence of text in a panel increases the likelihood that readers will visit that panel. Panels without text are more likely to be skipped on the first read-through (18).

However, the participants in these studies focus on eye movement lacked comics reading experience. Specifically, the study participants in the research by Tom Foulsham et al. (10) were mostly unfamiliar with comics, exhibiting a very low frequency of comic reading. As a multimodal text, comics involve both images and text, with panel layout and visual language elements. These factors differentiate comic reading from pure text reading. It is reasonable to speculate that comic reading indeed involves more complex visual and cognitive processing mechanisms.

Although the impact of the multimodal characteristics of comics on eye movement has not been explored, there are studies on eye movements exploring how readers simultaneously process multiple sources of information (text and images) in stimuli presented as a single item, such as music reading (33) and subtitles in films (2; 8). Wang and Jian (44) discussed how visual attention shifts between text and diagrams during science learning, analyzing the differences in visual processing between text and diagrams.

### Aim and research questions

In the medium of comics, words and images are combined to narrate a story through a sequence of panels. This study extends the investigation of comic reading. It particularly examines comic book readers' eye movements to enhance our understanding of visual literacy.

The study utilizes eye tracking to compare the viewing patterns of 14 experts (comic book enthusiasts with extensive reading experience) and 17 non-experts (individuals without experience in reading comic books) when reading comics. Differences in viewing patterns may indicate that experts have honed their visual reading skills over time.

Based on the expertise theory, we pose Research Question 1: In comic reading, do experts and non-experts exhibit significant differences in their viewing patterns? Consequently, we propose that experts and non-experts exhibit significant differences in fixations (H1a), and we expect to find significant differences in saccades between experts and non-experts (H1b).

As multimodal texts, comics involve different modalities in visual processing — text and images — a distinction not yet verified in traditional reading studies. Therefore, this study poses Research Question 2: In comic reading, does the content type (text versus images) affect the impact of expertise on viewing patterns? We hypothesize that there is an interaction between stimulus type and expertise regarding fixations and saccades (H2a and H2b). Specifically, since experts have greater expertise in visual language while both experts and novices have a relatively similar level of expertise in reading text, we expect that experts will examine the image part of the comic more thoroughly, and they may spend more time on it compared to non-experts.

## Methods

### Participants

Previous studies recruited visual experts, including visual communication professionals such as graphic designers, art and creative directors, and production artists (4), as well as students majoring in art (42), among others. However, in visual communication, there are significant differences between various fields such as painting, sketching, and graphic design. The 15 expert participants included comic book enthusiasts recruited through the university's anime clubs. The 20 non-expert participants included students at the university.

Convenience sampling was adopted to recruit 35 participants (male = 21, female = 14). None reported color blindness. All participants were native Chinese speakers with normal or corrected vision. In return for participating in the study, they received 20 yuan. They ranged in age from 18 to 28 years old, with one participant who was 30 years old.

Due to tracking ratios below 70%, four recordings were excluded from the analysis: 3 from non-experts and 1 from an expert. Consequently, the analysis primarily focused on the eye-tracking data of 14 experts and 17 non-experts. The age of experts (n = 14) was 23.35 ± 2.88 years, and the age of non-experts (n = 17) was 24.82 ± 2.87 years.

### Equipment

Participants' eye movements were monitored and recorded using the Tobii Pro Spectrum eye tracker. The eye tracker had a sampling rate of 1200 Hz. The device was connected to a 26-inch monitor with a resolution of 1920x1080 pixels. The stimuli were displayed on the screen at a size of 766 × 1080 pixels. The viewing distance was approximately 50-60 cm. All experiments were conducted in the same room.

### Materials

Previous research found that differences in viewing patterns only appear for images that are unfamiliar to the viewer. When viewing familiar artworks, the distinctions between experts and non-experts disappear (1; 11). Therefore, to avoid the influence of familiarity from repeated viewing on reading patterns, neither the novice nor expert groups had been exposed to the selected comics beforehand.

Four comics were selected, each containing complete plots. While we used only a small number of individual stories, our analyses were page-based and panel-based (Table 1); thus, the four stories provided a relatively large dataset for investigation. The four comics used were Doraemon (Fujiko F. Fujio); Fairy Cat (Takano Hisa, 2023); Ame to Kimi to (Nikaidou Ko, 2020); and Please Take My Brother Away! (You Ling, 2016).

The study did not manipulate the comic material. Instead, our aim was to collect data on natural reading behavior with these stimuli.

**Table 1.** Comic Page Panel and Text Information.

Comic	Panel Count	Textless Panel Count	Is Vertical Layout	Speech Bubble Count	Word Count	Has Blockage
Comic1-p1	5	0	Z-path	8	42	No
Comic1-p2	5	0	Z-path	7	46	No
Comic1-p3	6	0	Z-path	8	69	No
Comic1-p4	4	0	Z-path	5	43	Yes
Comic2-p1	6	3	Z-path	4	20	Yes
Comic2-p2	5	1	Z-path	4	23	No
Comic2-p3	3	2	Z-path	1	11	No
Comic2-p4	3	2	Vertical	1	8	No
Comic2-p5	6	1	Z-path	7	81	No
Comic3-p1	3	2	Z-path	2	2	No
Comic3-p2	4	0	Z-path	4	24	No
Comic3-p3	3	0	Z-path	0	0	No
Comic3-p4	5	4	Z-path	1	9	Yes
Comic3-p5	5	4	Z-path	5	9	Yes
Comic3-p6	5	2	Z-path	3	13	Yes
Comic3-p7	2	1	Vertical	3	16	No
Comic4-p1	4	2	Vertical	5	50	No
Comic4-p2	4	2	Vertical	3	19	No
Comic4-p3	4	1	Vertical	4	12	No
Comic4-p4	4	3	Vertical	3	15	No
Comic4-p5	3	0	Vertical	5	39	No
Comic4-p6	4	0	Vertical	6	23	No
Comic4-p7	3	0	Vertical	5	22	No

### Procedure

Each test session lasted 12-15 minutes. Following a calibration procedure, the participant was presented with the comic pages. We used the built-in calibration tool provided by Tobii eye-tracking software. The calibration process involved positioning participants, tracking calibration points on the screen, verifying accuracy, and recalibrating if necessary. The calibration error was within 0.4 degrees of visual angle, ensuring the accuracy and reliability of the eye-tracking data.

Participants could "flip" to a new page by clicking the mouse. They were informed that it was an open-ended (i.e., without time restriction) and free exploration task, where they were encouraged to read the comics for as long as they wished and to turn the pages as naturally as possible. After reading the comics, participants were asked to fill out a VLFI scale.

### Variables

#### Expertise Level

Expertise Level was assessed via the Visual Language Fluency Index (VLFI) of Cohn (7) to measure the participants' proficiency in comic reading. The VLFI questionnaire asked participants to rate on a scale of 1-7 their frequency of reading across two periods: current and their childhood (16 years old and younger). They were also asked to rate current and childhood expertise in drawing and comic book reading. The VLFI scores range from 1 to 52.5, with higher numbers indicating better comic fluency.

Based on the scores of the VLFI scale, participants were divided into two groups. Therefore, the variable Expertise Level has two levels, namely "Expert" and "Non-Expert".

The results showed that the average score of the participants was 13.82 (SD = 11.17). The average score of expert participants (n = 14) was 23.5 (SD = 9.18), while the mean score of non-experts participants (n = 17) was 5.85 (SD = 3.99). The results of an independent sample T-test (Table 2) showed that there were significant differences in VLFI between the two groups (t = -5.67, p < 0.001).

**Table 2.** Independent Samples T-Test Results for VLFI.

	*Mean*	*SD*	*t*	*p*
Non-experts (*n* = 17)	5.85	3.99	-5.67	< 0.001
Experts (*n* = 14)	23.5	9.18		

#### Content Type of AOIs

The variable Content Type was categorized as text and image. We divided the stimuli into areas of interest (AOI), primarily limited to specific panels (excluding their surrounding white areas such as gutters and page borders). We selected panels that contained narrative elements such as text and characters and recorded the Content Type of the AOIs. This allowed us to observe and compare the content type of attention.

### Covariates

To focus on the variables Expertise Level and Content Type, we controlled influence of the layout and text quantity on visual patterns (29; 15). We recorded each page's Panel Count, Textless Panel Count, Is Vertical Layout, Has Blockage, Speech Bubble Count, and Word Count. These variables served as control variables in our regression analysis.

Both Panel Count and Textless Panel Count were numerical variables; A Textless Panel was defined as one lacking any textual elements, including onomatopoeia or symbols, with higher values indicating greater visual content. Is Vertical Layout and Has Blockage were categorical variables assessing layout complexity. According to Cohn & Campbell (7), it is essential to consider these variables as they distinguish five types of Comic Page Layouts and analyze constraints on reading order. We measured layout using whether it is a Vertical structure or the "Z-path" (other layouts were minimally represented in our material). In the "Z-path" layout, blockages are common, where a long vertical panel obstructs the Z-path, directing readers to move vertically rather than horizontally (7). HB measured whether layouts use blockages. Speech Bubble Count and Word Count were numerical variables quantifying textual elements on the page.

#### observed variable

The study produced one set of data from the eye tracker. The data of eye tracker included Average duration of whole fixations, Number of whole fixations, Average amplitude of saccades, and Number of saccades (Table 3).

**Table 3.** Description of Observed Variables.

Metric name	Description
*Average duration of whole fixations*	The Average duration of the fixations inside an AOI or a page.
*Number of whole fixations*	The number of fixations occurring in an AOI or a page.
*Average amplitude of saccades*	The total amplitude of all saccades in an AOI or a page.
*Number of saccades*	The number of saccades occurring in an AOI or a page.

## Data Analysis

The Linear mixed model regression provides a more advanced level of analysis, allowing for the estimation of both fixed effects and random effects (28). Generalized linear mixed models (GLMMs) are an extension of linear mixed models, allowing response variables to come from different distributions (35). In this study, all participants were exposed to all stimuli, resulting in repeated measures. Additionally, it is expected that participants' responses to stimuli from the same comic are correlated. The number of fixations and the number of saccades follow a Poisson distribution, while the average duration of whole fixations and the average amplitude of saccades are positively skewed continuous data, suitable for a Gamma distribution. Therefore, it is necessary to adopt GLMMs, incorporating both participants and comics as random effects in the model.

The analysis was divided into two parts. The first part analyzed the effects of Expertise Level, while the second part focused on the Areas of Interest (AOIs), analyzing the effects of Expertise Level and Content Type, as well as their interaction.

### The viewing patterns between experts and non-experts

Except for the Average Duration of Whole Fixations, all other variables did not follow a normal distribution. Therefore, we used a Generalized Linear Mixed Model (GLMM) to determine whether there were statistically significant differences in viewing patterns between experts and non-experts while controlling for page layout and the amount of text. We employed the lmer4 library in R. The comic was entered as a random factor to account for its associated correlation. Then, we added the main predictor, Expertise Level, as well as the covariates. To improve the model fit, we standardized the control variables. This helped eliminate differences in scale among variables, thereby enhancing the stability of the model parameter estimates.

**Table 4.** Descriptive analysis of the dependent variable.

	Experts (*M* ± *SD*) (*n* = 14)	Non-experts (*M* ± *SD*) (*n* = 17)	Overall (*M ± SD*)
*Number of whole fixations*	21.32 ± 12.20	22.58 ± 14.99	22.08 ± 13.96
*Average duration of whole fixations* (ms)	219.21 ± 41.15	205.90 ± 43.57	211.16 ± 43.09
*Number of saccades*	19.45 ± 12.03	18.75 ± 13.60	19.03 ± 13
*Average amplitude of saccades* (°)	4.69 ± 1.47	4.40 ± 1.72	4.52 ± 1.63

**Table 5.** Effects of Expertise Level on Number of whole fixations and Average duration of whole fixations.

	Number of whole fixations ^a^			Average duration of whole fixations (ms) ^b^		
	B	*SE*	*z value*	B	*SE*	*t value*
**(Intercept)**	3.03	0.09	33.15***	206.97	9.08	22.80**
*Expertise Level*	-0.01	0.03	-0.08	14.17	10.77	1.32
**covariates**						
*Panel Count*	-0.02	0.04	-0.61	-1.64	2.42	-0.68
*Textless Panel Count*	0.16	0.03	5.95***	9.59	1.80	5.32***
*Is Vertical Layout*	-0.15	0.04	-3.89	3.17	2.56	0.21
*Has Blockage*	-0.03	0.02	-1.35	-4.16	1.54	-2.71**
*Speech Bubble Count*	0.19	0.04	5.00***	-6.38	2.52	0.012*
*Word Count*	0.12	0.03	3.91***	10.001	2.12	4.73***
Random effect		Variance	*SD*		Variance	*SD*
*Intercept：comic*		0.032	0.178		149.1	12.21
N _comic_		4			4	
Observations		678			678	
AIC		4782.7			6561.237	
BIC		4827.9			6610.948	
logLik		2381.4			-3269.619	
Marginal R^2^		0.312			0.079	
Conditional R^2^		0.431			0.578	

Note. a Generalized linear mixed-effects model fit by MLE; Link Function: Log; family: Negative Binomial. b Linear mixed model fit by REML. Reference category for *Expertise Level* (Expert = 1, Non-expert = 0). Reference category for *Is Vertical Layout* (Vertical = 1, Z-path = 0). Reference category for *Has Blockag*e (Yes =1, No = 0). *** p < .001, ** p < .05

**Table 6.** Effects of Expertise Level on Number of saccades and Average amplitude of saccades.

	Number of saccades ^a^			Average amplitude of saccades (°) ^b^		
	B	*SE*	*z value*	B	*SE*	*t value*
**(Intercept)**	2.85	0.09	30.96***	1.46	0.02	86.63***
*Expertise Level*	0.09	0.04	2.20*	0.08	0.03	2.95**
**covariates**						
*Panel Count*	-0.03	0.04	-0.61	-0.08	0.03	-2.91**
*Textless Panel Count*	0.17	0.03	5.05***	-0.02	0.02	-0.84
*Is Vertical Layout*	-0.14	0.05	-3.04**	-0.08	0.02	-4.54**
*Has Blockage*	-0.0	0.03	-1.09	0.01	0.02	0.58
*Speech Bubble Count*	0.20	0.05	4.47***	0.02	0.03	0.64
*Word Count*	0.12	0.04	3.19**	-0.09	0.02	-3.55***
Random effect		Variance	*SD*		Variance	*SD*
*Intercept：comic*		0.032	0.178		149.1	12.21
N _comic_		4			4	
Observations		678			678	
AIC		4821.13			2453.78	
BIC		4866.33			2498.97	
logLik		-2400.56			-1216.89	
Marginal R^2^		0.25			0.15
Conditional R^2^		0.35			NA	

Note. a Generalized linear mixed-effects model fit by MLE; Link Function: Log; family: Negative Binomial. b Generalized linear mixed-effects model fit by MLE; Link Function: Log; family: Gamma. Reference category for Expertise Level (Expert = 1, Non-expert = 0). Reference category for Is Vertical Layout (Vertical = 1, Z-path = 0). Reference category for Has Blockage (Yes =1, No = 0). *** p < .001, ** p < .05

The Mixed Linear Model and Generalized linear mixed-effects model (Table 5 and Table 6) were used to test if Expertise Level significantly predicted the *Average duration of whole fixations*, *Number of whole fixations*, *Average amplitude of saccades* and *Number of saccades*. Results revealed that Expertise Level significantly impacted the *Average amplitude of saccades* (B = 0.08, p = 0.003) and *Number of saccades* (B = 0.09, p = 0.028).

Expertise Level did not impact the *Average duration of whole fixations* (B = 14.17, p = 0.199) and *Number of whole fixations* (B = -0.01, p = 0.935). This implies that there were no significant differences in the Average duration of whole fixations between the experts (M = 219.21, SD = 41.15) and the non-experts (M = 205.90, SD = 43. 57). Similarly, no significant differences were observed in the Number of whole fixations between experts (M = 21.32, SD = 12.2) and non-experts (M = 22.58, SD = 14.99).

The experts exhibited a higher Average amplitude of saccades (M = 4.69, SD = 1.47) compared to the non-experts (M = 4.40, SD = 1.72), indicating greater saccadic movements. The number of saccades was greater in the expert group (M = 19.45, SD = 12.03) than in the non-expert group (M = 18.75, SD = 13.60).

H1a was not supported, while H1b was supported, demonstrating that the differences between experts and non-experts are evident in saccades rather than fixations. These findings suggest that expertise in comic reading is more strongly associated with the efficiency and speed of saccadic movements, rather than the duration or frequency of fixations. This highlights that experts may be better at quickly and effectively processing visual information through more efficient saccades.

### The viewing patterns between experts and non-experts in AOIs

In this study, a 2 (Expertise Level: expert vs. non-expert) × 2 (Content Type: image vs. text) mixed design was employed. Based on the division into Areas of Interest (AOIs), GLMM was conducted to discuss the differences in viewing patterns between experts and non-experts within these specific areas.

**Table 7.** Effects of Expertise Level and Content Type on Average duration of whole fixations and Number of whole fixations.

	Average duration of whole fixations (ms) ^a^			Number of whole fixations ^b^		
	B	*SE*	*t value*	B	*SE*	*z value*
**(Intercept)**	5.34	0.05	108.25***	0.37	0.13	2.86**
*Expertise Level*	0.03	0.06	0.54	-0.19	0.16	-1.20
*Content Type*	-0.11	0.02	-6.82***	0.15	0.03	5.35***
*Expertise Level* * *Content Type*	0.05	0.02	2.17*	0.36	0.05	7.85***
Random parts		Variance	*SD*		Variance	*SD*
*Intercept: comic*		0.001	0.021		0.030	0.173
*Intercept: participant*		0.005	0.075		0.171	0.414
N _comic_		4			4	
N _participant_		31			31	
Observations		3420^c^			5236	
AIC		38061.68			20163.01	
BIC		38104.64			20202.39	
logLik		-19023.84			-10075.5	
Marginal R^2^		0.028			0.041	
Conditional R^2^		0.085			0.343	

Note. a Generalized linear mixed-effects model fit by MLE; Link Function: Log; family: Gamma. b Generalized linear mixed-effects model fit by MLE; Link Function: Log; family: Poisson. c The AOIs with zero number of whole fixations are not included. Reference category for Expertise Level (Expert = 1, Non-expert = 0). Reference category for Content Type (Image = 1, Text = 0).

**Table 8.** Effects of Expertise Level and Content Type on Number of saccades in AOI.

	Number of saccades in AOI ^a^		
	B	*SE*	*z value*
**(Intercept)**	-0.52	0.18	-2.85**
*Expertise Level*	-0.34	0.22	-1.52
*Content Type*	-0.02	0.04	-0.48
*Expertise Level* * *Content Type*	0.60	0.07	8.39***
Random parts		Variance	*SD*
*Intercept: comic*		0.054	0.233
*Intercept: participant*		0.342	0.585
N _comic_		4	
N _participant_		31	
Observations		5236	
AIC		13425	
BIC		13464.38	
logLik		-6706.499	
Marginal R^2^		0.023	
Conditional R^2^		0.332	

Note. a Generalized linear mixed-effects model fit by MLE; Link Function: Log; family: Poisson. Reference category for Expertise Level (Expert = 1, Non-expert = 0). Reference category for Content Type (Image = 1, Text = 0).

For the Average duration of whole fixations (Table 7), the main effect of expertise level was not significant (B = 0.03, p = 0.59), while the main effect of content type was marginally significant (B = -0.11, p = 0.01). The interaction between expertise level and content type was significant (B = 0.05, p = 0.03).

For the number of whole fixations (Table 7), the main effect of expertise level was not significant (B = -0.18, p = 0.23), while the main effect of content type was significant (B = 0.15, p = 0.01). The interaction between expertise level and content type was significant (B = 0.36, p = 0.01).

For the number of saccades in AOI (Table 8), the main effect of expertise level was not significant (B = -0.34, p = 0.12), while the main effect of content type was not significant (B = -0.02, p = 0.63). The interaction between expertise level and content type was significant (B = 0.59, p = 0.01).

The results indicate that content type has a significant effect on fixations (Table 6), and there was a significant interaction between expertise level and content type. These findings support our hypotheses (H2a and H2b), suggesting that in comic reading, the interaction between different content types and expertise levels has a significant impact on viewing patterns.

**Figure 1.**
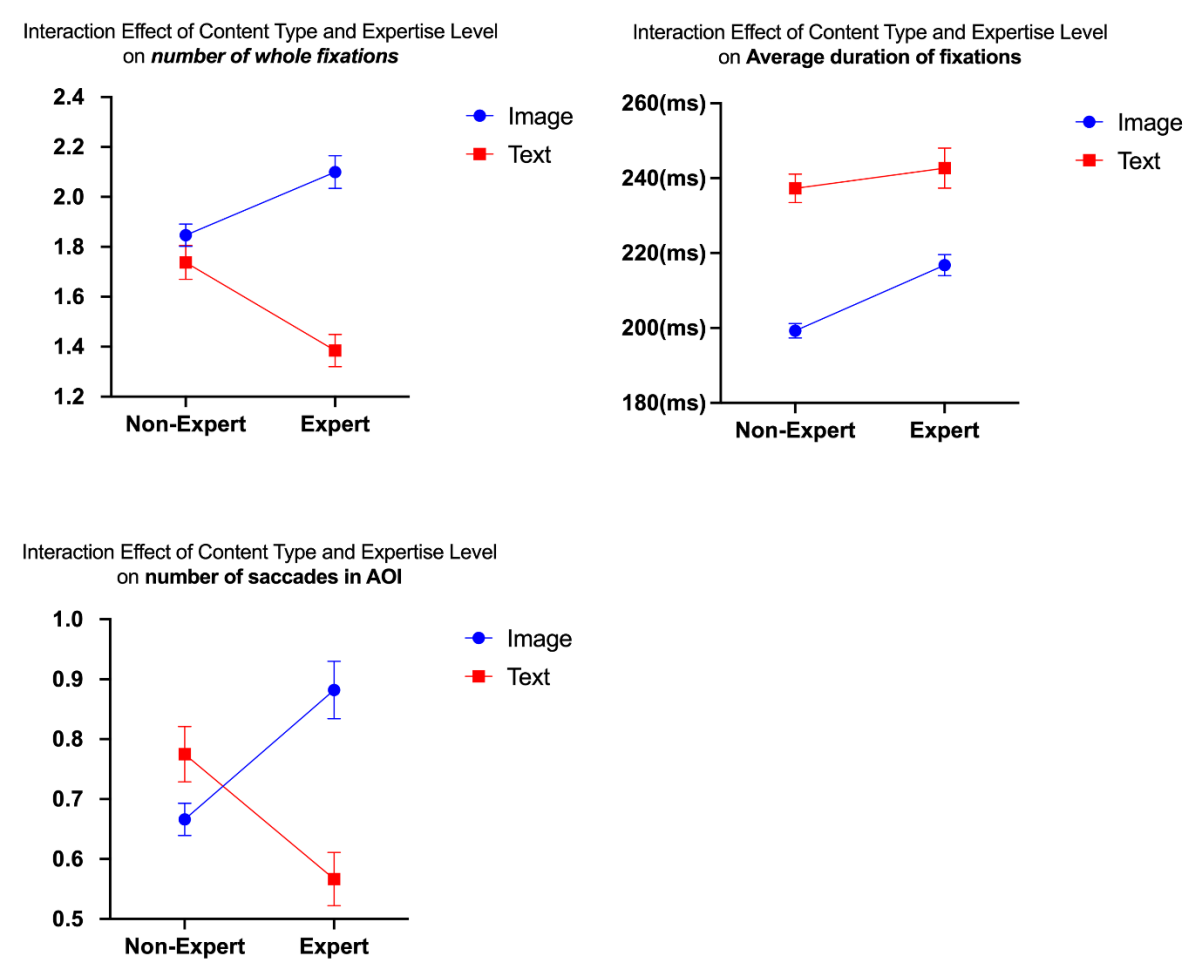
Interaction Effect of Content Type and Expertise Level.

Both non-experts and experts had significantly more fixations on text content (non-experts =1.74 ± 2.32, experts = 1.38 ±1.76) compared to images (non-experts = 1.85 ± 2.02, experts = 2.10 ± 2.36). However, the difference in the number of fixations between text and images was greater for experts than for non-experts. Both non-experts and experts also had longer fixation durations on text content (non-experts = 237.26 ± 102.66, experts = 242.68 ± 111.56) than on images (non-experts = 199.26 ±73.73, experts = 216.76 ± 88.08), with experts showing longer fixation durations on both content types than non-experts. Non-experts exhibited more saccades on text (0.78 ± 1.59) content than on images (0.67 ± 1.21), whereas experts had more saccades on image content (0.88 ± 1.72) than on text (0.57 ± 1.21).

## Discussion

### Main Findings

In this study, we used an eye-tracker to measure the comic reading behaviors of experts and non-experts and employed Generalized Linear Mixed Models (GLMMs) to analyze the effects of Expertise Level and Content Type on visual attention metrics. These metrics included the number of fixations, the number of saccades, the average duration of fixations, and the average amplitude of saccades. We explored whether there were differences in comic reading between experts and non-experts. Additionally, by defining specific Areas of Interest (AOIs), we further examined the interaction between Expertise Level and Content Type.

The results of the GLMM analysis indicated that, after controlling for variables such as layout and text, expertise level had a significant impact on saccade amplitude. Compared to non-experts, experts have larger saccades. This supports our hypothesis (H1b).

Previous studies have found that a high number of words and a greater percentage of word occupancy in panels were associated with longer dwell times, along with character-focused images (17). This study found a main effect of content type on several visual measurement metrics. Non-experts and experts differed significantly in the number of saccades and the average amplitude of saccades (H1), whereas differences in the number of fixations and the average duration of fixations across expertise levels were not significant. This could be due to the relatively weak influence of expertise level on visual attention compared to other factors such as comic page layout and the amount of text. As von Wartburg et al. (43) confirmed, saccade amplitudes vary with image size.

Significantly, the interaction between expertise level and content type had a notable impact on several AOI-based visual metrics. This supports our hypotheses (H2a and H2b) and extends previous research. It reveals that experts and non-experts respond differently to images and text. Experts showed greater attention to images, with more fixations and larger saccades compared to text. In contrast, non-experts required longer fixation times and more fixations when reading text. This suggests that experts process text more efficiently, possibly due to their experience with integrating textual and visual information, a skill honed through expertise in visual information processing.

In terms of fixation duration on text, there is not much difference between experts and non-experts. However, compared to non-experts, experts exhibit longer fixation durations and a greater number of fixations on images. Similar differences are observed in saccadic: experts have more saccades when viewing images, while non-experts have more saccades when reading text. This suggests that, although both experts and non-experts are skilled readers, experts place greater emphasis on the visual aspects of comics. This may be due to their specific expertise in visual language, which provides them with more experience in integrating text and visual information.

These differences indicate distinct visual scanning strategies between non-experts and experts, especially when processing different types of content. Experts tend to exhibit higher levels of activity and efficiency in image processing, while non-experts appear to exert more effort but with lower efficiency in text processing. Experts are inclined to search for underlying patterns rather than concentrating only on the most prominent feature (i.e., text). Our results suggest that cognitive processes and visual attention mechanisms differ significantly between experts and non-experts. Experts' greater attention to images and more efficient processing of text content can be attributed to their enhanced ability to integrate textual and visual information, a skill honed through extensive practice and experience. This supports the notion that expertise leads to a dominance of top-down processing over bottom-up processing, as evidenced by Fudali-Czyż et al. (12).

### Theoretical Significance

This study provides support for the expertise theory in the field of comic reading. It found significant differences in visual scanning behavior between experts and non-experts when processing text and image content, particularly in terms of strategies and efficiency in visual processing. Specifically, experts showed higher speeds when processing image content compared to non-experts. These findings offer a new perspective on how expertise level influences visual information processing and further validate the applicability of expertise theory in comic reading.

Previous studies have indicated that a high number of words and a greater percentage of word occupancy in panels are associated with longer dwell times, as well as character-focused images (17). Our study extends this understanding by demonstrating that expertise level interacts with content type, impacting several AOI-based visual metrics. Experts showed more fixations and larger saccades for images compared to non-experts, whereas non-experts exhibited more saccades and more fixations when reading text. This suggests that experts paying more attention to the visual aspects of comics, consistent with findings in other visual domains (19; 14).

### Contrasting Findings

Interestingly, some findings of this study differ from Zhao & Mahrt (46), who reported that experienced readers had shorter fixations than inexperienced readers in comic reading. In our study, the difference in fixation duration between non-experts and experts was not significant. This discrepancy may be due to our inclusion of control variables such as layout and text quantity, which previous studies have shown significantly impact eye movements (29; 15).

### Future Work

To further understand the multimodal nature of comics, we intend to conduct more in-depth analyses. Firstly, we aim to analyze reading sequences under the "Z" layout and investigate skipping (failure to look at the next panel in the sequence) and regression (looking back to an earlier panel in the sequence) behaviors. Additionally, we have not yet classified panels based on the integration of text and images, which could be an area for future exploration.

Moreover, our study found that expertise had no significant effect on the number of fixations but did have an effect on the number of saccades. This difference in the coefficient might be due to the distinct cognitive processes underlying these two types of eye movements. While both fixations and saccades provide valuable insights into visual information processing, they may be differentially influenced by expertise. However, our experimental design did not initially anticipate this specific focus, and therefore, we did not conduct a deeper analysis of these differences. Given these findings, future research could further explore this aspect to better understand how expertise shapes the cognitive processes involved in visual information processing.

### Ethics and Conflict of Interest

The author declares that the contents of the article are in agreement with the ethics described in http://biblio.unibe.ch/portale/elibrary/BOP/jemr/ethics.html and that there is no conflict of interest regarding the publication of this paper.

### Acknowledgements

The author would like to thank Han Jiaxiang for his help in performing the experiment.

The author is grateful to the anonymous reviewers for their valuable comments and suggestions, which have significantly contributed to the improvement of this manuscript.
